# Pharmacokinetics (PK), pharmacodynamics (PD), and PK-PD integration of ceftiofur after a single intravenous, subcutaneous and subcutaneous-LA administration in lactating goats

**DOI:** 10.1186/s12917-016-0863-9

**Published:** 2016-10-13

**Authors:** Emilio Fernández-Varón, Carlos Cárceles-García, Juan Manuel Serrano-Rodríguez, Carlos M. Cárceles-Rodríguez

**Affiliations:** 1 Department of Pharmacology, Faculty of Veterinary Medicine, University of Murcia, Campus de Espinardo, 30.071, Murcia, Spain; 2 Department of Pharmacology, Toxicology and Legal and Forensic Medicine, Faculty of Veterinary Medicine, University of Cordoba, Campus de Rabanales, 14.071, Córdoba, Spain

**Keywords:** Ceftiofur, Pharmacokinetic/Pharmacodynamic, Long-acting formulation, Lactating goats

## Abstract

**Background:**

Bacterial pneumonia in goats is usually caused by *Mannheimia haemolytica* and *Pasteurella multocida.* Another important infection disease in lactating goats is intramammary infection producing mastitis, usually associated with coagulase-negative *Staphylococcus* spp. However, treatment of bacterial pneumonia in goats not affected by mastitis problems should be restricted to antimicrobials with scant penetration to milk in order to avoid long withdrawal times. Ceftiofur is a third-generation cephalosporin antimicrobial with activity against various gram-positive and gram-negative, aerobic and anaerobic bacteria encountered by domestic animals. The objectives of the present study were to establish the serum concentration–time profile for ceftiofur in lactating goats after intravenous, subcutaneous and a SC-long-acting ceftiofur formulation; to determine ceftiofur penetration into milk; to determine in vitro and ex vivo activity of ceftiofur establishing MIC, MBC, MPC and time-kill profiles against field strains of *M. haemolytica* and finally to calculate the main surrogate markers of efficacy.

**Results:**

The pharmacokinetics studies revealed an optimal PK properties for the SC-LA formulation tested. Ceftiofur was well absorbed following SC and SC-LA administration, with absolute bioavailabilities (F) of 85.16 and 84.43 %, respectively. After ceftiofur analysis from milk samples, no concentrations were found at any sampling time. The MIC, MBC and MPC data of ceftiofur against five *M. haemolytica* strains isolated from goats affected by pneumonia were tested showing excelent sensitivity of ceftiofur against this pathogen. For PK-PD analysis, ratios were calculated suggesting a high level of bacterial kill against the five strains of *M. haemolytica* tested.

**Conclusions:**

The systemic ceftiofur exposure achieved in lactating goats following IV, SC and especially with the SC-LA administration is consistent with the predicted PK-PD ratios needed for a positive therapeutic outcome for *M. haemolytica*. Subcutaneous administration of the long-acting formulation showed safety and tolerance for all the animals used. Ceftiofur concentrations exceeded the MIC and MBC for up to 72 h and MPC for up 32 h in serum. Thus, this drug could be effective in treating infectious diseases of goats caused by *M. haemolytica* at a dose of 6 mg/kg with the SC-LA formulation.

**Electronic supplementary material:**

The online version of this article (doi:10.1186/s12917-016-0863-9) contains supplementary material, which is available to authorized users.

## Background

Ceftiofur is a third-generation cephalosporin antimicrobial with activity against various gram-positive and gram-negative, aerobic and anaerobic bacteria encountered by domestic animals [1]. As with all cephalosporins, it is considered bactericidal because it inhibits bacterial cell wall synthesis and its pharmacokinetic and pharmacodynamic (PK ⁄ PD) parameter most associated with efficacy is the time above a threshold concentration, typically the MIC [2].

Over the past years, the food and milk that goats provide have become more popular in Europe and in the United States [3]. Bacterial pneumonia are frecuent in this species, and the most common bacterial isolates from lungs of goats affected by pneumonia are *Pasteurella multocida* and *Mannhemimia haemolytica* [3, 4]. Another important infection disease in lactating goats is intramammary infection producing mastitis, usually associated with coagulase-negative *Staphylococcus* spp. However, treatment of bacterial pneumonia in goats not affected by mastitis problems should be restricted to antimicrobials with scant penetration to milk in order to avoid long withdrawal times. Other antimicrobials agents that could be used in pneumonia are fluoroquinolones and macrolides that have a wide milk penetration.

Ceftiofur has been synthesized in various salt forms. The sodium and hydrochloride salts were first developed and approved worldwide for treatment of respiratory disease in beef and dairy cattle. Additional indications were also approved in swine, goats, sheep, horses, day-old chickens, day-old turkey poults and dogs [5]. The third salt approved was a ceftiofur free acid form in a sterile oil formulation (CCFA) showing excellent sustained-release properties. This oily, non-aqueous suspension of CCFA has good efficacy and has been studied in different animal species like horses [6], cattle [5], lactating and non-lactating goats [3]. However, issues with residues, inflammatory reactions and pain at the injection site have been reported.

In the present study, we have assayed a subcutaneous, aqueous, polymeric sustained-release formulation with the sodium salt. This subcutaneous long-acting formulation has been made with polymer P407. P407 is a block of copolymers contains 7 % polyoxyethylene units and 30 % polyoxypropylene units. This polymer has some advantages as its low toxicity, excellent compatibility with other chemicals and a high solubilizing capacity for different drugs. P407 shows temperature-dependent gelation (gel consistency at 37 °C and liquid consistency at 4 °C) [7]. The P407 gel has been evaluated for the delivery of biologically active proteins such urease and interleukin-2 [8, 9] and antibiotics as ceftiofur [7], doxycycline [10], moxifloxacin [11] and difloxacin [12].

The objectives of the present study were: (1) to establish the serum concentration–time profile and to derive pharmacokinetics data for ceftiofur in lactating goats after intravenous (IV), subcutaneous (SC) and a subcutaneous long-acting (SC-LA) ceftiofur formulation; (2) to determine the rate and extent of ceftiofur penetration into and elimination from milk after IV, SC and SC-LA administration of ceftiofur; (3) to determine in vitro activity of ceftiofur establishing MIC and MBC in serum and Mueller Hinton broth, and MPC of ceftiofur for five strains of *Mannheimia haemolytica* isolated from caprine (4) to determine ex vivo time-kill profiles of ceftiofur against the five strains of *M. haemolytica* in both in serum and Mueller-Hinton broth (5) to calculate the surrogate markers of efficacy against *Mannheimia haemolytica* strains isolated from goats affected by pneumonia.

## Methods

### Animals

Six clinically healthy Murciano-Granadina female lactating goats weighing between 40.5 and 56 kg and aged from 2.5 to 3.5 years from the Caprine Farm of the University of Murcia were used. The animals were housed and fed an antibiotic-free diet for at least 30 days preceding the study. For each treatment period of the cross-over study, they were observed daily for general health, and clinical observations were made prior to injection and at 2, 10 and 24 h post-injection. Alfalfa hay and water was provided *ad libitum* together with a drug-free concentrate. The study was approved by the Bioethics Committee of the University of Murcia.

### Experimental design

A cross-over design (2 × 2 × 2) was used in three phases. Each animal received either a single IV or SC injection of ceftiofur sodium (Excenell® 4 g, Pfizer, Madrid, Spain) at a dose of 2.2 mg/kg or a SC-LA administration of 6.6 mg/kg with at least a 15-day washout period.

For the IV administration, the solution was injected into the left jugular vein and blood samples (4 mL) were collected from the contralateral jugular vein. SC and SC-LA injections were administered under the skin of the back at a single location in the thoraco-lumbar region lateral of the mid-line. Blood samples were collected at 0 (pre-treatment), 0.083, 0.167, 0.25, 0.5, 0.75, 1, 1.5, 2, 4, 6, 8, 10, 12, 24, 32, 48, 72, 96 and 120 h post-dosing. Samples were centrifuged at 1500 g for 15 min and the serum taken and stored at −90 °C until assayed.

Milk samples for analysis were collected from each goat after complete evacuation of the udder by manual stripping of each gland immediately before dosing on the day of treatment administration (time 0) and at 1, 2, 4, 6, 8, 10, 12, 24, 32, 48, 72, 96 and 120 h after administration. After shaking the milk to homogenize, a 4–5 mL sample was collected and stored at −90 °C until assayed.

### Gel preparation

Gel was prepared on a weight basis using the cold method [13]. Concentrations of P407 and ceftiofur reported here are expressed as weight percentage (% wt/wt). An amount of P407 sufficient to yield 25 % and Carboxymethylcellulose sodium to yield 2 % gel was slowly added at 4 °C and ceftiofur sodium sufficient to yield a 20 % concentration was dissolved in the cold solution.

### Analytical method

Serum and milk samples were analyzed for concentrations of ceftiofur, desfuroylceftiofur, and related metabolites by reduction and derivatization to desfuroylceftiofuracetamide (DCA) [14]. Ceftiofur and desfuroylceftiofur-related metabolites were extracted from serum and milk following a reduction step through the addition of 1,4-dithioerythritol solution (20 mg/mL in 0.1 M ammonium acetate, pH = 8.9). An internal standard consisting of a cefotaxime solution (200 μg/mL) was incorporated in this step. Following a 30-min incubation step at 50 °C to fully reduce the thioester bond in ceftiofur and desfuroylceftiofur-related metabolites, the resulting desfuroylceftiofur was captured on a C18 solid-phase extraction columns (Oasis HLB SPE cartridges, Waters, Barcelona, Spain) and further derivatized with iodoacetamide to create desfuroylacetamide (DCA). DCA was removed from the column with 30:70 acetonitrile: 0.01 M ammonium acetate with 0.1 % trifluoroacetic acid providing a final injection equal to isocratic HPLC conditions (15 % acetonitrile: 85 % 0.01 M ammonium acetate (0.1 % trifluoroacetic acid)). The HPLC separation was performed using a reverse-phase Kinetex ^TM^ PFP C18 column (250 × 4.6 mm; 5 μm) with an injection volume of 100 μL. Ultraviolet detector was set at 240 nm.

### Method validation

Quality controls were prepared from a pool of blank goat serum or milk spiked with seven concentrations of ceftiofur between 0.10 and 10 μg/mL. Serum and milk aliquots were stored at −90 ° C until assay. Aliquots of quality controls were extracted as above and 100 μL was injected into the chromatographic system. Standard curves were obtained by unweighted linear regression of ceftiofur and cefotaxime peak areas versus known concentrations. Each point was established from an average of five determinations. Correlations coefficients (r) were >0.99 for calibration curves. The percentage recovery was determined by comparing the peak areas of serum and milk blank samples spiked with different amounts of drug and treated as any samples, with the peak areas of the same standards prepared in phosphate buffer. Each point was established from an average of five determinations. The mean percentage recoveries of ceftiofur from serum and milk were 84.79 and 88.16 %, respectively. The assay precision (R.S.D.) was assessed by expressing the standard deviation of repeated measurements as a percentage of the mean value. Serum intra-day precision was estimated from six replicates of three standard samples used for calibration curves (R.S.D. < 5.83 %). Milk intra-day precision was R.S.D. < 10.2 %. Inter-day precision was estimated from the analysis of standard samples (serum or milk) on three separate days. Serum inter-day obtained a R.S.D. < 4.91 %. Milk inter-day assay obtained a R.S.D. < 6.54 %. The limit of quantification (LOQ) and the limit of detection (LOD) for was 0.1 μg/mL for serum and milk.

### Bacterial strains, MIC, MBC and MPC determination

Five field strains of *M. haemolytica* isolated from goats affected by pneumonia in Spain were used. The strains were stored at −80 °C in a nutrient broth enriched with 15 % glycerol until assayed. The MIC of ceftiofur was determined in goat serum and Mueller-Hinton broth (MHB, Fluka analytical, Madrid, Spain) using the microdilution method recommended by CLSI [15]. The assay was performed in U-bottomed, 96-well, custom-designed microtiter plates. After overnight incubation at 37 °C on tryptone soya blood agar plates, bacterial suspensions equal to a 0.5 McFarland standard were further diluted and 10 μL were added to the plates to achieve a final inoculum of 5 × 10^5^ CFU/mL. The plates contained antimicrobial dilutions ranging from 0.03 to 128 μg/mL in 90 μL/well of goat serum or MHB, the final volume was 100 μL. Assays were incubated at 37 °C and observed after 24 h. The MIC was taken as the lowest drug concentration that inhibited visible growth. MBC was established by plate count as the concentration of antibacterial to reduce a 3log_10_ (99.9 % killing) the initial inoculum, in accordance with CLSI guidelines [15].

The MPC was measured by agar dilution using a method previously described [16]. Briefly, the content of overnight cultures of each strain of *M. haemolytica* (5 plate per isolate) in TSBA was transferred to 100 mL of MHB and incubated overnight at 37 °C with shaking at 200 rpm. The next day, bacterial suspensions were estimated to have concentrations close to 3 · 10^8^ CFU/mL by turbidity measurements. Then, cultures were concentrated by centrifugation at 5000 g for 30 min at 5 °C and re-suspended in 3 mL of fresh MHB. Aliquots of 200 μL containing 10^10^ CFU were inoculated in TSBA plates previously prepared with ceftiofur over a range from 0.03 to 128 μg/mL. Plates were incubated for 48 h at 37 °C and screened visually each 24 h for growth. The MPC was recorder as the lowest concentration that prevented the growth of colonies.

For MIC, MBC and MPC assays, a non-inoculated plate was included as a negative control, and an inoculated plate without drug as a positive control. Also, *S. aureus* ATCC 29213 and *E. coli* ATCC 25922 strains were used as controls. All determinations were performed in duplicate, and the geometric mean was calculated.

### In vitro antimicrobial growth (time-kill) curves

Time kill curves were obtained in a second trial after MIC, MBC and MPC determination. In vitro and ex vivo activity of ceftiofur against *M. haemolytica* was obtained using a method previously described [17]. Eight to 10 colonies from overnight cultures in TSBA of each strain were used to inoculate 9 mL of MHB and incubated overnight at 37 °C with shaking at 200 rpm.

For in vitro assays, 480 μL of MHB or goat serum were spiked with 10 μL of concentrated solutions of ceftiofur. The final samples contained antimicrobial concentrations at 0 (control), 0.25, 0.5, 1, 2, 4, 8 and 16 multiples of MIC previously obtained in each fluid. A total of 10 μL of stationary-phase bacterial culture was added to give a final concentration of approximately 5 · 10^6^ CFU/ml. Aliquots of 25 μL were sampled from each culture at 0, 1, 2, 4, 8 and 24 h. Counts were determined by serial dilution in saline and spread on TSBA agar plates [18]. For ex vivo testing, serum samples from goat which had received ceftiofur IV, SC or SC-LA at 2.2 or 6.6 mg/kg respectively, were collected at 0, 1, 2, 4, 12, 24, 32, 48, and 72 h. Concentrations of ceftiofur in these samples were previously determined with the HPLC method described in the above section. Then, a total of 10 μL of stationary-phase bacterial culture was added, and aliquots of 25 μL were sampled from each culture at 0, 1, 2, 4, 8 and 24 h using the same methodology described for in vitro time kill curves. Counts were determined by serial dilution in saline and spread on TSBA agar plates. The lower limit of detection was 40 CFU/mL for both in vivo and ex vivo assays. Time kill curves were performed at 37 °C with shaking at 200 r.p.m.

### Pharmacokinetic analysis

The serum ceftiofur time-concentration data were analysed by non-compartment methods using WinNonLin 5.2 (Pharsight Corp, Mountain View, Calif). The area under the concentration-time Curve (AUC) was calculated using the linear trapezoidal rule with extrapolation to time infinity. Mean Residence Time was calculated as MRT = AUMC/AUC. Mean absorption times were calculated as MAT = MRT_SC, SC-LA_ – MRT_IV_ and K_a_ was calculated non-compartment methods as K_a_ = 1/MAT. The systemic clearance was estimated as Cl = Dose/AUC. The apparent volume of distribution at steady state were calculated as V_ss_ = (Dose · AUMC)/AUC^2^. The apparent volume of distribution (area method) was calculated as V_z_ = Dose/ (AUC · λ_z_). Biovailability (F) was calculated by the method of corresponding areas:

$$ \left[\mathrm{F}\left(\%\right) = \left({{\mathrm{AUC}}_{\mathrm{SC}}}_{,\ \mathrm{S}\mathrm{C}\hbox{-} \mathrm{L}\mathrm{A}}\cdotp\;{\mathrm{Dose}}_{\mathrm{IV}}\right)\times 100\ /\ \left({\mathrm{AUC}}_{\mathrm{IV}}\cdotp\;{\mathrm{Dose}}_{\mathrm{SC},\ \mathrm{S}\mathrm{C}\hbox{-} \mathrm{L}\mathrm{A}}\right)\right] $$

### Statistical analysis

Descriptive statistical parameters as mean, standard deviation and coefficient of variation were calculated. Harmonic means were calculated for the half-lives of elimination. A Kruskal-Wallis analysis of variance, followed by a Dunn multiple comparison test if applicable was used to test parameters for significant differences between IV, SC and SC-LA administration. Dose-dependent (AUC and C_max_) parameters were corrected with dose to test for statistical differences. The level of significance was P ≤ 0.05. The statistical software used was SPSS (IBM SPSS Statistics, Version 19.0. Armonk, NY: IBM Corp).

## Results

**Fig. 1:**
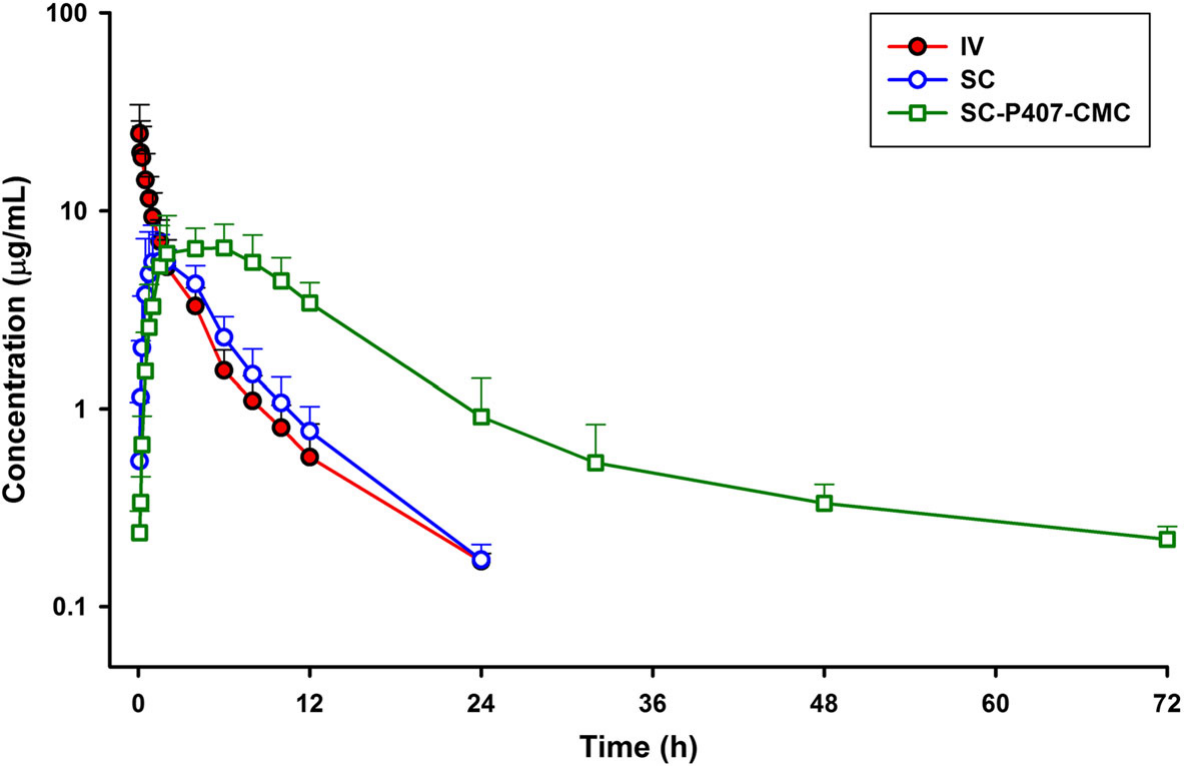
Semilogarithmic plot of serum concentrations (mean ± SD) of ceftiofur and desfuroylceftiofur-related metabolites after IV administration (2.2 mg/kg) (IV; -●-), SC administration (2.2 mg/kg) (SC; -○-), and SC-LA administration (6.6 mg/kg) (SC-LA; -□-) in goats (*n* = 6)

The mean (±SD) serum concentrations of ceftiofur following IV, SC and SC-LA administration are plotted in Fig. 1. The estimates of the non-compartmental pharmacokinetics parameters following IV, SC and SC-LA administration are summarized in Table 1.

**Table 1:** Pharmacokinetic parameters (mean ± S.D.) of ceftiofur in lactating goats after IV and SC administration at a dose of 2.2 mg/kg bodyweight, and after SC-LA administration at a dose of 6.6 mg/kg bodyweight (*n* = 6)

Treatment			
Parameters	Intravenous	SC	SC-LA
λ _z_ (1/h)	0.16 ± 0.07	0.14 ± 0.06	0.02 ± 0.01 ^a, b^
t_½λz_^1^ (h)	4.21	5.10	41.12^a, b^
V_ss_ (L/kg)	0.18 ± 0.05	--	--
V_z_ (L/kg)	0.31 ± 0.14	--	--
AUC_0–24_ (μg · h/mL)	45.51 ± 6.80	38.17 ± 6.97	85.13 ± 7.53 ^a^
AUC_0-∞_ (μg · h/mL)	47.06 ± 6.84	39.88 ± 6.26	117.12 ± 11.54 ^a^
MRT (h)	4.27 ± 0.98	6.29 ± 0.35^a^	25.11 ± 4.35 ^a, b^
Cl (L/h · kg)	0.04 ± 0.01	--	--
MAT (h)	--	2.02 ± 1.09	20.83 ± 5.13 ^b^
K_a_ (1/h)	--	0.53 ± 0.49	0.03 ± 0.01 ^b^
C_max_ (μg/L)	--	6.25 ± 0.85	7.77 ± 1.26
T_max_ (h)	--	0.91 ± 0.20	4.66 ± 2.05 ^b^
F (%)	--	85.16 ± 10.24	84.43 ± 7.40

T_½λz_: The elimination half-life associated with the terminal slope (λ_z_) of a semilogarithmic concentration-time curve. V_z_: The apparent volume of distribution calculated by the area method. V_ss_: The apparent volume of distribution at steady state. AUC_0-∞_: The area under the serum concentration-time curve from zero to infinity. AUC_0–24_: the area under the serum concentration–time curve from zero to 24 h. MRT: Mean residence time. MAT: Mean absorption time. Cl: The total body clearance of drug. T_max_: The time to reach peak concentration following extravascular administration. MAT: Mean absorption time. K_a_: Absorption rate constant. C_max_: The peak concentration following extravascular administration. F: The fraction of the administered dose systemically available (bioavailability). ^a^Significantly different from IV (*P* < 0.05). ^b^Significantly different from SC (*P* < 0.05). ^1^ Harmonic Mean

Clinical examination of all goats after each phase of the trial did not reveal any abnormalities. Local or systemic adverse reactions were not observed neither during nor after IV, SC and SC-LA administration, respectively.

The ceftiofur half-life (t_½λz_) for IV, SC and SC-LA routes was 4.21, 5.10 and 41.12 h. Clearance value after IV dosing was 0.04 ± 0.01 L/kg•h. After SC and SC-LA administration, the absolute bioavailability was mean (±S.D) 85.16 ± 10.24 % and 84.43 ± 7.40 %, respectively. The C_max_ was 6.25 ± 0.85 μg/mL (SC) and 4.26 ± 0.68 μg/mL (SC-LA) with a T_max_ of 0.91 h (SC) and 4.66 h (SC-LA). There were no significant differences between values calculated for C_max_, AUC_0-∞_ and AUC_0–24_ for both subcutaneous routes of administration (dose corrected for comparison). However, significant differences (*P* < 0.05) were found between SC and SC-LA formulations for λ_z_, t_1/2λz_, MRT, MAT, K_a_ and T_max_ as expected.

After ceftiofur analysis from milk samples, no concentrations were found at any sampling time.

**Fig. 2:**
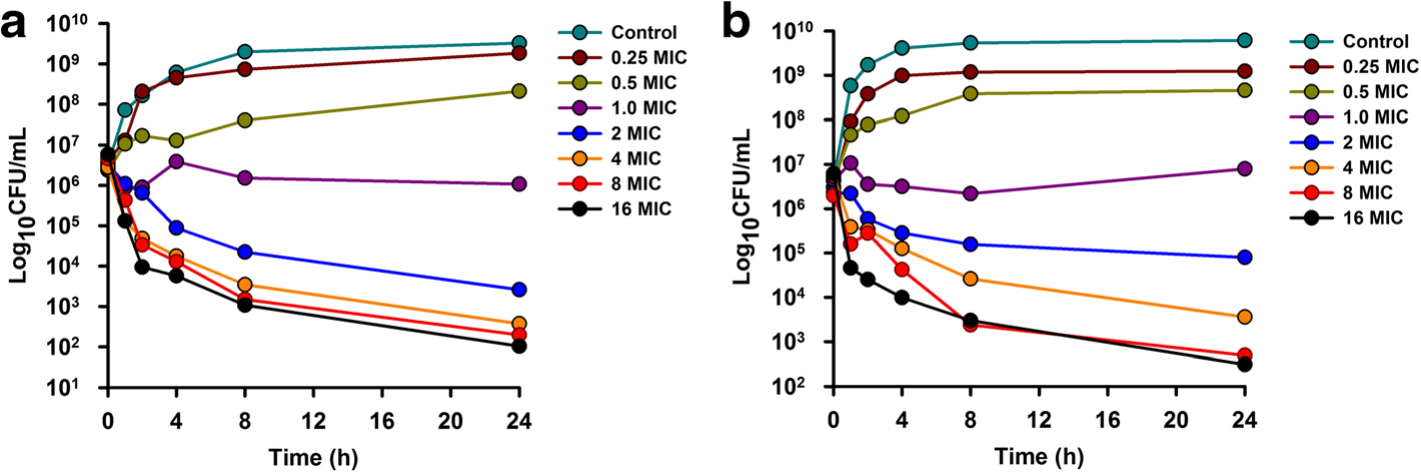
In vitro antibacterial activity of ceftiofur in MHB (**a**) and serum (**b**) of goats against *M. haemolytica* expressed as mean values (*n* = 5). SD values are excluded for clarity

**Fig. 3:**
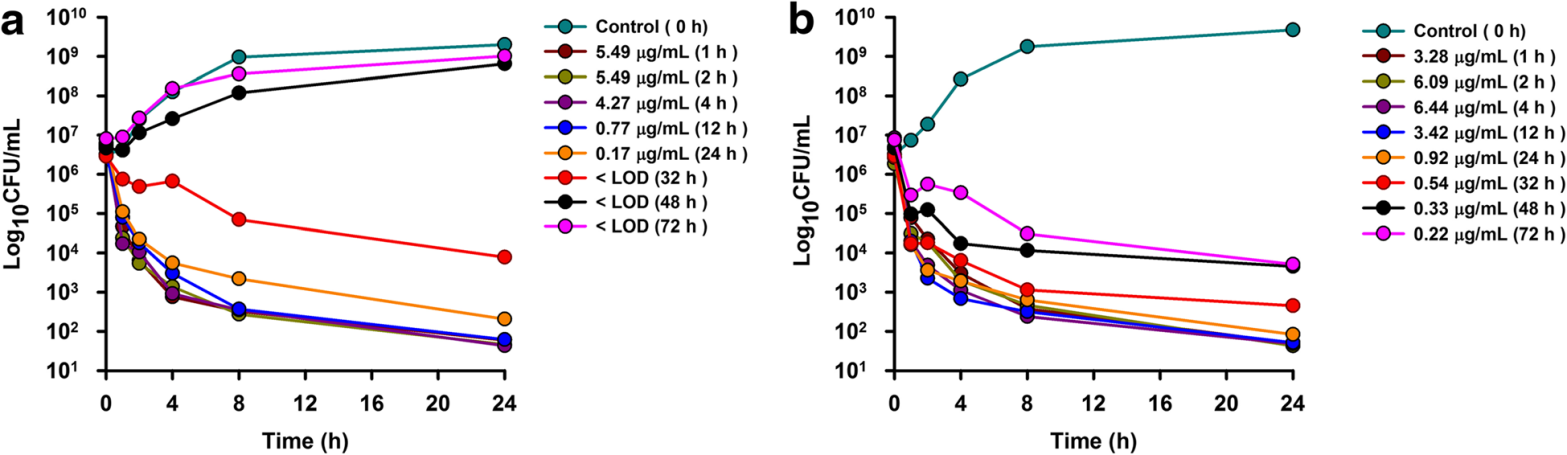
Ex vivo antibacterial activity of ceftiofur from 1 to 72 h in (**a**) goat serum after SC administration at a dose of 2.2 mg/kg bodyweight, and (**b**) goat serum after SC-LA administration at a dose of 6.6 mg/kg bodyweight against *M. haemolytica*, values are mean (*n* = 5). SD values are excluded for clarity

The MIC, MBC and MPC values were obtained from strains of *M. haemolytica* isolated from goats affected by pneumonia. Ceftiofur showed excellent in vitro activity, the surrogate markers were calculated and are shown in Table 2. Time-kill curves in serum and Mueller-Hinton broth are shown in Fig. 2. Time-kill curves in goat serum after SC and SC-LA administration of ceftiofur are shown in Fig. 3.

**Table 2:** Surrogate markers of efficacy from pharmacokinetics parameters after IV, SC and SC-LA administraton of ceftiofur, calculated for a mean MIC, MBC and MPC of ceftiofur on *M. haemolytica* strains isolated from goats (*n* = 5)

Parameter	IV	SC	SC-LA
Mean MIC (*M. haemolytica*) = 0.0436 μg/mL			
C_max_/MIC	--	130.53 ± 28.65	145.47 ± 22.55
AUC_0-∞_/MIC (h)	1079.16 ± 157	914.77 ± 143	2686.34 ± 264
AUC_24_/MIC (h)	1043.80 ± 155	875.38 ± 160	1952.44 ± 173
T > MIC (h)	24 h	24 h	72 h
Mean MBC (*M. haemolytica*) = 0.10 μg/mL			
C_max_/MBC	--	56.91 ± 12.49	63.42 ± 9.83
AUC_0-∞_/MBC (h)	470.51 ± 68.46	398.84 ± 62.60	1171.24 ± 115.4
AUC_24_/MBC (h)	455.1 ± 67.98	381.66 ± 69.78	851.27 ± 75.36
T > MBC (h)	24 h	24 h	72 h
Mean MPC (*M. haemolytica*) = 0.55 μg/mL			
C_max_/MPC	--	10.34 ± 2.27	11.53 ± 1.79
AUC_0-∞_/MPC (h)	85.54 ± 12.36	72.51 ± 11.38	212.95 ± 20.98
AUC_24_/MPC (h)	82.74 ± 12.36	69.39 ± 12.69	154.77 ± 13.70
T > MPC (h)	12 h	12 h	32 h

## Discussion

The pharmacokinetics of ceftiofur sodium has been studied in goats [19], sheep [20], camels [21], llamas [22], deers [23], horses [6], fouls [14] and pigs [24]. However, there are limited data available in lactating animals and especially in small ruminants like lactating goats. Besides, to our knowledge there is no information about ceftiofur pharmacokinetics and pharmacodynamics integration data of the main pathogens involve in pneumonia in goats (*Pasteurella multocida* and *Mannheimia haemolytica*) and studies with long-acting formulations are very limited [7, 11, 12]. Some years after ceftiofur sodium development and commercial availability, a new sustained-release ceftiofur salt was developed as crystalline-free acid (CCFA). The long-acting formulation has been studied in different animal species as horses [6], cattle [5], lactating and non-lactating goats [3]. Importance of development of sustained-release formulations in veterinary medicine and especially in food animal species has been increasing last years. The advantages of long-acting formulations of ceftiofur (and other drugs) in lactating goats include less quantity of drug used (decreasing collateral effects and accumulation in long-term treatments), increased treatment efficacy (less fluctuations of the stationary concentrations and much longer release times) and a reduction in handling (stress of the animals and veterinary costs are decreased). On the other hand, it is necessary to consider drug safety, efficacy and residues at the site of infection as serious issues with these formulations. Although most studies have concluded that ceftiofur free-acid in pigs and cattle was well tolerated systemically, frecuent adverse effects have been reported at the injection sites with the CCFA formulation. Other formulations have been studied like liposomes in cows [25]. In the present study an aqueous polymeric sustained-release formulation has been developed for subcutaneous administration. After IV administration, the half-life (t_½λz_ = 4.21 h) was slightly higher than that reported in lactating goats after IV dosing of ceftiofur sodium (t_½λz_ = 3.88 h) [19]_,_ and clearance (Cl = 0.04 L/h · kg) was lower than the data reported by the same authors (0.08 L/h · kg). The volume of distribution (0.31 and 0.18 L/kg calculated by the area method (V_z_) and at steady-state (V_ss_), respectively, suggests limited penetration through biological membranes, and these values indicate that ceftiofur is widely distributed into the extravascular tissues. In other studies, larger but also limited volume of distribution V_ss_ was described for ceftiofur sodium in lactating (0.31 L/kg) and non-lactating (0.25 L/kg) goats [19]. Elimination half-life (t_½λz_) of the SC-LA formulation (41.12 h) was far longer than that for the SC administration (5.1 h) showing prolonged permanence of the drug in serum with this long-acting formulation. Ceftiofur was well absorbed following SC and SC-LA administration, with absolute bioavailabilities (F) of 85.16 and 84.43 %, respectively. MAT was 2.02 h for the SC administration and ten-fold longer for the SC-LA formulation (20.83 h). Absorption rate constant (K_a_) was 0.53 h^−1^ for the SC administration and 0.03 h^−1^ after SC-LA. These absorption data reflect that the polymeric formulation has reduced considerably the rate of absorption affecting the elimination rate where the SC-LA formulation showed a significantly longer elimination half-life respect to the conventional SC formulation.

After ceftiofur analysis from milk samples, no concentrations were found at any sampling time. This result is in agreement with other studies in goats [3] and cows [26]. Ceftiofur sodium has a pK_a_ value of 3.7. Ceftiofur sodium in the blood stream (pH 7.4) would act as a weak acid with and insufficient lipid-soluble properties at this pH to penetrate milk, which can also explain limited volume of distribution obtained.

Pharmacodynamics of ceftiofur against *M. haemolytica* in MHB showed in vitro maximum growths from 3.5°10^9^ to 7°10^9^ CFU/mL obtained at 24 h without drug. Ceftiofur concentrations multiples to 1 × MIC produced a 0log_10_ reduction in count over 24 h (bacteriostatic effect), whereas concentrations less than 1 × MIC produced no growth inhibition (Fig. 2a). A 3-4log_10_ reduction of count was achieved after 24 h from 2 × MIC (bactericidal effect). After in vitro time kill curves in serum, maximum growths from 4.4°10^9^ to 8.5°10^9^ CFU/mL were obtained at 24 h without drug. Concentrations less than or equal to 2 × MIC produced no growth inhibition with a 0-2log_10_ reduction in count over 24 h. A 3-4log_10_ reduction was achieved at concentrations similar or higher than 4 × MIC after 24 h of incubation, and a regrowth was not observed (Fig. 2b).

Ex vivo time kill curves showed antibacterial activity of ceftiofur against *M. haemolytica* in serum of lactating goats administered as a conventional SC and SC-LA formulation. The time points selected for the study with serum samples were of 0 (control), 1, 2, 4, 12, 24, 32, 48 and 72 h after drug administration. Maximum growths closed to 5°10^9^ CFU/mL were obtained at 24 h without drug. For SC time kill curve (Fig. 3a), a 3-4log_10_ reduction after 24 h incubation was achieved with ceftiofur samples obtained from 1 to 24 h. A 2 log_10_ reduction was observed with at samples measured at 32 h. However serum samples from 48 to 72 h produced no growth inhibition, whereas these time points achieved a 3log_10_ reduction after 24 h of incubation with the SC-LA formulation. Moreover, samples between 1 and 32 h produced an elimination of organisms from 8 to 24 h of incubation with a 4-5log_10_ reduction. These results could be explained by the ceftiofur concentrations achieved with the SC-LA formulation.

The most important factor determining the efficacy of β-lactam antimicrobials such as ceftiofur is the amount of time that serum concentrations exceed the MIC of a given pathogen [27, 28]. However, this approach, almost unanimous in literature, is been questioned in some cases. In a recent PK/PD study of a depot formulation of amoxicillin in calves against bovine respiratory pathogens, *Mannheimia haemolytica* and *Pasteurella multocida,* it was stated that for sustained-release formulations a concentration-dependent action would better fit according to the time-kill data obtained [29]. Similar results were found in sheep against *Mannheimia haemolytica* and *Pasteurella multocida* isolates for amoxicillin [30]. Additionally, it has been reported that for drugs like the β-lactams, where efficacy has been found to be correlated to the T > MIC, the best PK/PD index shifts towards AUC/MIC dependence as half-life increases [31]. In the present study, in vitro and ex vivo time-kill curves against *Mannheimia haemolytica* also suggest a possible concentration-dependent action. Therefore, the modelling of AUC/MIC data as surrogate for/predictor of efficacy is justified at least for these respiratory tract pathogens [32, 33]. The longer half-life obtained in the present study for the SC-LA formulation (41.12 h) regard to the SC (5.1 h) reinforce this approach for PK/PD integration of our data. Thus, PK/PD integration was calculated for the five strains of *M. haemolytica* using the three commonly used surrogates markers of efficacy, AUC/MIC, C_max_/MIC and T > MIC, required to inhibit growth.

The MIC, MBC and MPC data of ceftiofur against five *M. haemolytica* strains isolated from goats affected by pneumonia were tested. The MIC values in this study showed good sensitivity of ceftiofur against this pathogen. MIC, MBC and MPC are values tested in in vitro conditions, and it is known influence of matrix when MIC and MBC values are calculated (MHB and serum) mainly by effect of drug protein binding that should correspond to lower MIC and MBC values in MHB regard to serum values. In our study no differences were found in MIC values between both matrices. However in the case of MBC values calculated both in MHB and serum, differences were found in two strains (two-fold) between MHB and serum which is in agreement with the lower total protein concentrations present in MHB respect to serum [34].

On the other hand, although MIC values are the standard to calculate the three most important surrogate markers of efficacy C_max_/MIC, AUC_0–24_/MIC ratios and T > MIC to get an optimal dosage regimen, MIC values are considered less appropriate in preventing the emergence of resistant strains. The use of MPC values defined as the lowest drug concentration that prevents the growth of the least susceptible first-step resistant mutants have been considered to calculated AUC_24_/MPC ratios and the mutant selection window (MSW) that serve as an indicator of drug exposure that prevents the selection of drug-resistant mutant [35, 36]. In the present study, AUC_24_/MPC ratio and T > MPC were calculated and for the SC-LA formulation values of 154 h and 32 h were obtained, respectively, showing the good profile of the long-acting formulation to achieve both clinical efficacy and prevention of emergence of resistant strains. Besides, the MIC and MPC are also useful to define the bounds of the mutant selection window (MSW), a range of antibacterial concentrations in which drug least susceptible cells could be selected most frequently. MSW in this study range from 0.0436 and 0.55 μg/mL according to the mean MIC and MPC values. Concentrations of ceftiofur with the LA formulation exceed the MPC for the first 48 h which suggest that susceptible strains of *M. haemolytica* in vivo could be eradicated. However, it is necessary to be cautious with these results and to consider factors like in vitro conditions of MPC calculation, lack of studies, scores of the clinical symptoms in affected animals and the low number of strains tested in this study.

For PK-PD analysis, the MIC data of ceftiofur against *M. haemolytica* strains isolated from goats affected by pneumonia were tested resulting a mean MIC of 0.0436 μg/mL. The C_max_/MIC, AUC_24_/MIC, AUC_0-∞_/MIC ratios and T > MIC were calculated (Table 2). The four ratios used suggest a high level of bacterial kill against the five strains of *M. haemolytica* tested. For the SC-LA formulation AUC_24_/MIC, even AUC_0-∞_/MIC exceed widely 125 h, and C_max_/MIC exceed 10 as the ratios usually considered to have clinical efficacy. T > MIC was greater than 72 h for the SC-LA formulation. Although these ratios should be integrated with MIC distribution values from field isolates [29], these data show a high clinical efficacy against this pathogen and possibly against other pathogens in goats.

## Conclusions

The systemic ceftiofur exposure achieved in lactating goats following IV, SC and especially with the SC-LA administration is consistent with the predicted PK-PD ratios needed for a positive therapeutic outcome for *M. haemolytica*. Subcutaneous administration of the long-acting formulation showed safety and tolerance for all the animals used. Milk penetration of ceftiofur was nonexistent in all animals at least at the limit of detection of our study, which is a clear advantage in lactating animals affected by pneumonia avoiding withdrawal periods. This study has shown that ceftiofur concentrations exceeded the MIC and MBC for up to 72 h and MPC for up 32 h in serum. Thus, this drug could be effective in treating infectious diseases of goats caused by *M. haemolytica* at a dose of 6 mg/kg with the SC-LA formulation. However, further studies in affected animals taking into account other factors as scores of the clinical symptoms, to optimize and to know the optimal ratios of the surrogate markers of efficacy.

## Additional file

Additional file 1: Plasma Ceftiofur concentrations and time kill curves data. (XLSX 15 kb)

## Abbreviations

HPLC: High performance liquid chromatography
IV: Intravenouos
MBC: Minimum bactericidal cocentrations
MIC: Minimum inhibitory concentrations
MPC: Mutant prevention concentrations
PK-PD: Pharmacokinetics-pharmacodynamics
SC: Subcutaneous
SC-LA: Subcutaneous-Long acting
